# Identification of Differently Expressed Genes Associated With Prognosis and Growth in Colon Adenocarcinoma Based on Integrated Bioinformatics Analysis

**DOI:** 10.3389/fgene.2019.01245

**Published:** 2019-12-04

**Authors:** Ming Hu, Xiandong Fu, Zhaoming Si, Chunming Li, Jihu Sun, Xinna Du, Hu Zhang

**Affiliations:** ^1^Department of General Surgery, First Affiliated Hospital of Jiamusi University, Jiamusi, China; ^2^Department of Proctology, Jiamusi Central Hospital, Jiamusi, China; ^3^Department of Physiology and Biochemistry, Jiangsu Vocational College of Medicine, Yancheng, China

**Keywords:** colon adenocarcinoma, differentially expressed genes, survival, fitness gene, bioinformatics

## Abstract

Latest statistics showed that the morbidity and mortality of colon adenocarcinoma (COAD) ranked fourth and fifth, respectively, around the world. COAD was a heterogeneous disease, and the high rates of recurrence, metastasis, and drug resistance still posed great challenges for treatment, which needs to further develop therapeutic and prognostic targets. In this study, we got the top 3,075 differentially expressed genes (DEGs) and 1,613 potential prognostic genes by GEPIA 2 and identified 1,166 fitness genes in COAD based on genome-scale CRISPR-Cas9 knockout (GeCKO) screening data. Excluding the genes already reported in the literatures, a total of nine DEGs overlapping with prognostic and fitness genes were further analyzed. High expression of *CCT6A*, *RHOQ*, and *RRP12* promoted COAD cell growth and were relative to lower survival rate of COAD patients, while high expression of *UTP18*, *DDOST*, *YRDC*, *ACTG1*, *RFT1*, and *NLE1* also promoted COAD cell growth, but were relative to higher survival rate. In addition, *CCT6A*, *UTP18*, *YRDC*, *RRP12*, *RFT1*, *NLE1*, as well as *DDOST* were essential genes across pan-cancer including COAD cells, and *ACTG1* and *RHOQ* were less essential genes in cancer cells. In a word, we discovered nine novel potential genes that could serve as anticancer targets and prognostic markers in COAD and its subtypes.

## Introduction

Malignant tumors have become one of the major public health problems that seriously threaten the health of people all over the world. Global cancer morbidity and mortality were still rising year by year. It was estimated that there would be 18.1 million new cancer cases worldwide in 2018, and the death toll would reach 9.6 million (Bray et al., 2018). Comparing with the 2012 statistical report (about 14.1 million new patients, 8.2 million died of cancer) (Torre et al., 2015), new cancer cases in 2018 had increased by nearly 30% and deaths had increased by 17%. The incidence and mortality of colon adenocarcinoma (COAD) were ranked fourth (6.1%) and fifth (5.8%), respectively (Bray et al., 2018). As the most populous country in Asia and the world, China ranked first in the world in terms of new cancer and deaths (Jindong 2018; Rongshou et al., 2019). According to the report in 2019, there were about 3.929 million cases of cancer in China. The incidence was 285.83/100,000, the annual death was about 2.338 million, and the mortality was 170.05/100,000 in 2015 (Rongshou et al., 2019). Among them, colorectal cancer (CRC) incidence and mortality ranked third and fifth, respectively. It could be seen that the global burden of cancer including COAD was increasing, and cancer was about to become the leading cause of human death in the 21st century.

CRC, including colon and rectal cancer, was one of the most common gastrointestinal tumors. CRC usually originated from benign adenomatous polyps, gradually developed into adenomas with high dysplasia, and eventually progressed to invasive cancer. Although early surgery and postoperative chemotherapy could effectively treat CRC, the high rates of recurrence, metastasis, and drug resistance still posed great challenges for the treatment of CRC (Aran et al., 2016; Favoriti et al., 2016; Hu et al., 2016; Kandagatla et al., 2018). It was urgent to further develop therapeutic and prognostic targets due to the heterogeneity (Pan et al., 2016; Kandagatla et al., 2018) and poor prognosis of CRC (Favoriti et al., 2016; Tang et al., 2019).

In the past decade, with the development of high-throughput and multi-omics data as well as the accumulation of clinical data of tumors, new potential pathogenic genes and prognostic markers had been discovered in tumors including COAD (Rhodes et al., 2004; Cancer Genome Atlas 2012; Robinson et al., 2017; Ghandi et al., 2019). However, the results tended to be variant because of different sample sizes and sources, control groups, or statistical methods. In addition, the recently emerging genome-scale CRISPR-Cas9 knockout (GeCKO) library screening technology provided a large number of potential genes related to tumor cell proliferation and metastasis (Chen et al., 2015; Behan et al., 2019; Chan et al., 2019; Chow et al., 2019). In this study, we identified differentially expressed genes (DEGs) associated with the prognosis and growth of COAD based on integrated bioinformatics analysis.

## Materials and Methods

### Identification of DEGs Based on TCGA and the GTEx Data

Gene expression profiling interactive analysis (GEPIA) is a web-based tool to mine and understand gene functions in tumors based on RNA-Seq data from TCGA and GTEx (Tang et al., 2017), and GEPIA 2 () is an updated and enhanced version of GEPIA, which records 275 COAD and corresponding 41 normal samples of TCGA as well as 308 colon samples of GTEx. DEGs in COAD were identified by matching TCGA normal and GTEx data through GEPIA 2. ANOVA was used for tumor vs. paired normal samples.

### Identification of Prognostic Genes Based on TCGA Data

In addition to DEGs, identification of genes with the most significant association with patient survival is another important use of GEPIA 2. To identify genes that affect OS or RFS (disease-free survival) in COAD, patient populations were split into two groups by median or quartile expression (high vs. low expression). Finally, four prognostic gene lists were put together as potential prognostic genes of COAD.

### Functional Enrichment Analysis

GSEA was used to conduct enrichment analysis of gene expression data in COAD and normal samples (Subramanian et al., 2005). Hallmark gene sets, KEGG, and oncogenic signatures were chosen as gene sets databases. “log_2_ Ratio of Classes” was chosen as “metric for ranking genes”. False discovery rate (FDR) q-val <0.05 was set as the cutoff criteria.

Gene ontology (GO) was performed for the top DEGs overlapping with potential prognostic genes and KEGG enrichment analysis by DAVID Bioinformatics Resources 6.8 (https://david.ncifcrf.gov/). FDR < 0.05 was defined as the cutoff criteria.

The results of functional enrichment analysis by GSEA and DAVID Bioinformatics Resources 6.8 were visualized by ImageGP (http://www.ehbio.com/ImageGP/). 

### Fitness Genes in COAD Mining From GeCKO Screening Data

Genome-scale CRISPR-Cas9 screening was performed on 324 human cancer cell lines from 30 cancer types by Behan and colleagues (2019). The gene fitness scores of the COAD cell lines, processed data, and results were downloaded from the project Score web portal (https://score.depmap.sanger.ac.uk). In the results, log_2_FC < −1 and FDR value <0.05 were set as the cutoff criteria. Those genes promoting growth of more than two cell lines were put together as fitness genes in COAD.

### Identification of Target Genes and Their Expression in COAD and Subtypes

The fitness genes overlapping with potential prognostic and top DEGs were genes of interest for further research. According to whether the microsatellite is stable or not, COAD can be divided into three subtypes: microsatellite instability—high (MSI-H), microsatellite instability—low (MSI-L), and MSS. Expression of target genes in COAD and subtypes were analyzed by GEPIA 2, which recorded 52 MSI-H, 52 MSI-L, and 184 MSS COAD and corresponding 41 normal samples of TCGA as well as 308 colon samples of GTEx. |Log_2_FC| cutoff was set as 1, *q* value cutoff was 0.01, and tumor and normal colors were set as red and black, respectively.

### Analysis of Prognostic Significance of Target Genes in COAD and Subtypes

Similarly, the OS or RFS of every target gene in MSI-H, MSI-L, or MSS COAD patients were analyzed by GEPIA 2. Group cutoff was set as median or quartile.

### The Effect of Target Genes on COAD Cell Line Growth

The effects of target gene depletion on COAD cell growth or viability were mined from GeCKO screening data downloaded from the project Score web portal (https://score.depmap.sanger.ac.uk), which contains 10 COAD cell lines. Log_2_FC < −1 indicated cell growth or viability was blocked by gene depletion.

### Pan-Cancer Analysis of Target Genes Dependency Score by DepMap

DepMap is a cancer dependency map that systematically identifies genetic and pharmacologic dependencies and the biomarkers that predict them (Tsherniak et al., 2017). The target genes dependency scores were analyzed by DepMap web portal () based on datasets of CRISPR (Avana) Public 19Q2. A lower CERES score indicates a higher likelihood that the gene of interest is essential in a given cell line. 0 indicates the gene is not essential and −1 is comparable to the median of all pan-essential genes (red line) (Meyers et al., 2017).

## Results

### DEGs in COAD and Functional Enrichment Analysis

**Figure 1:**
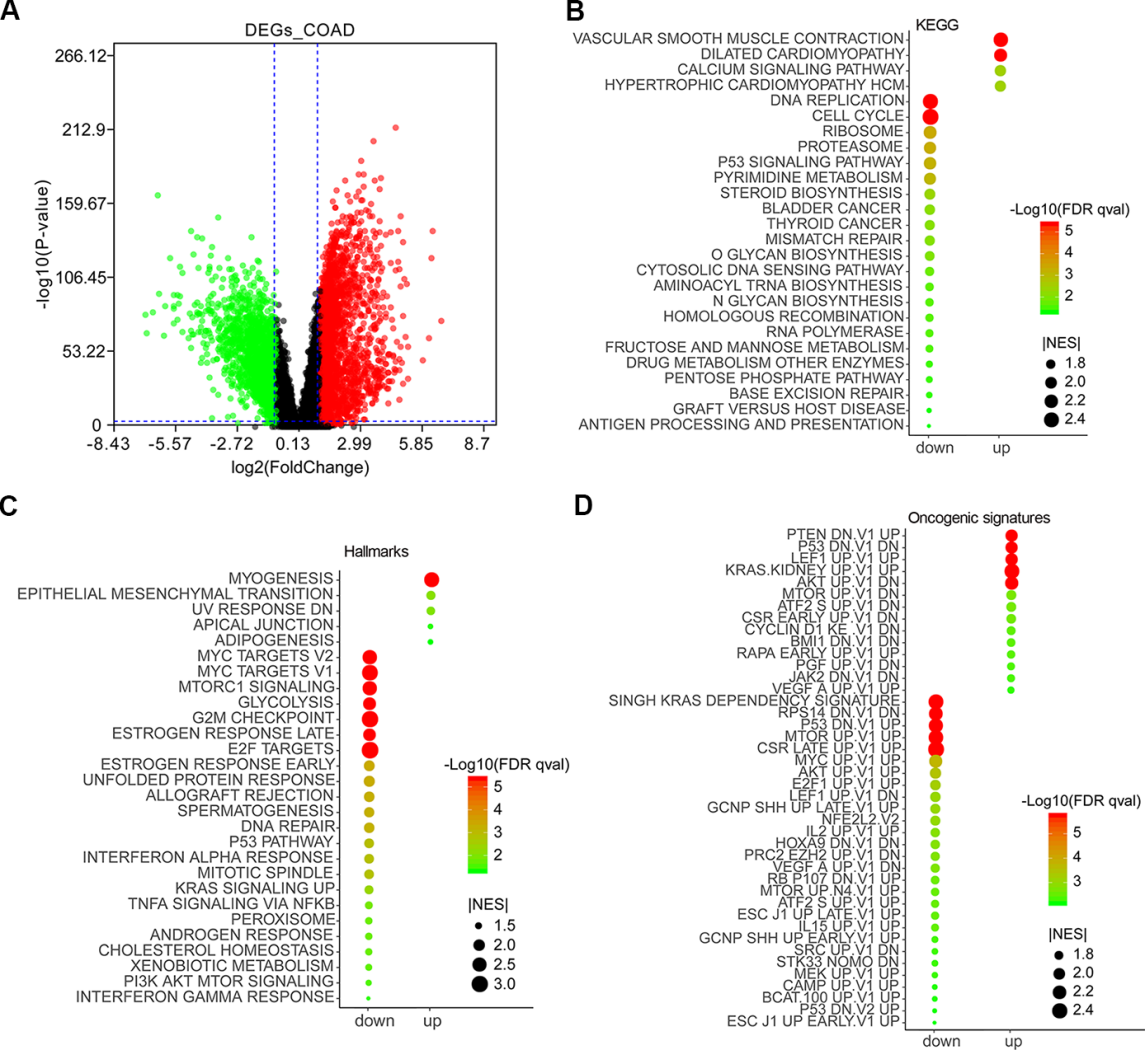
Differentially expressed genes (DEGs) in colon adenocarcinoma (COAD) and functional enrichment analysis by gene set enrichment analysis (GSEA). DEGs in COAD patients were mined by GEPIA 2 (http://gepia2.cancer-pku.cn/#analysis). *Red* and *green scatter* represented upregulated and downregulated genes, respectively **(A)**. Enrichment analyses of KEGG **(B)**, Hallmarks **(C)**, and oncogenic signatures **(D)** were conducted by GSEA and visualized by ImageGP (http://www.ehbio.com/ImageGP/).

The expression data of 27,043 genes in COAD and normal samples was performed enrichment analysis by gene set enrichment analysis (GSEA) (**Supplementary Table 1**). Except for lower expression genes, a total of 3,075 top DEGs containing 1,232 downregulated and 1,843 upregulated genes were obtained by GEPIA 2 (**Supplementary Table 1**) and were visualized as a volcano plot (**Figure 1A**). GSEA results showed that the downregulated genes were mainly enriched in DNA replication, cell cycle, ribosome pathway, etc., and the upregulated genes mainly enriched in vascular smooth muscle contraction, dilated cardiomyopathy, calcium signaling pathway, etc. (**Figure 1B**). As for Hallmarks results, the downregulated genes were mainly enriched in MYC targets, MTORC1 signaling, glycolysis, etc., and the upregulated genes mainly enriched in myogenesis, epithelial mesenchymal transition, adipogenesis, etc. (**Figure 1C**). According to oncogenic signature results, the downregulated genes were mainly enriched in SINGH KRAS DEPENDENCY SIGNATURE, RPS14 DN.V1 DN, P53 DN.V1 UP, etc., and the upregulated genes mainly enriched in PTEN DN.V1 UP, P53 DN.V1 DN, CYCLIN D1 KE V1 DN, etc. (**Figure 1D**).

### Potential Prognostic Genes in COAD and Functional Enrichment Analysis

**Figure 2:**
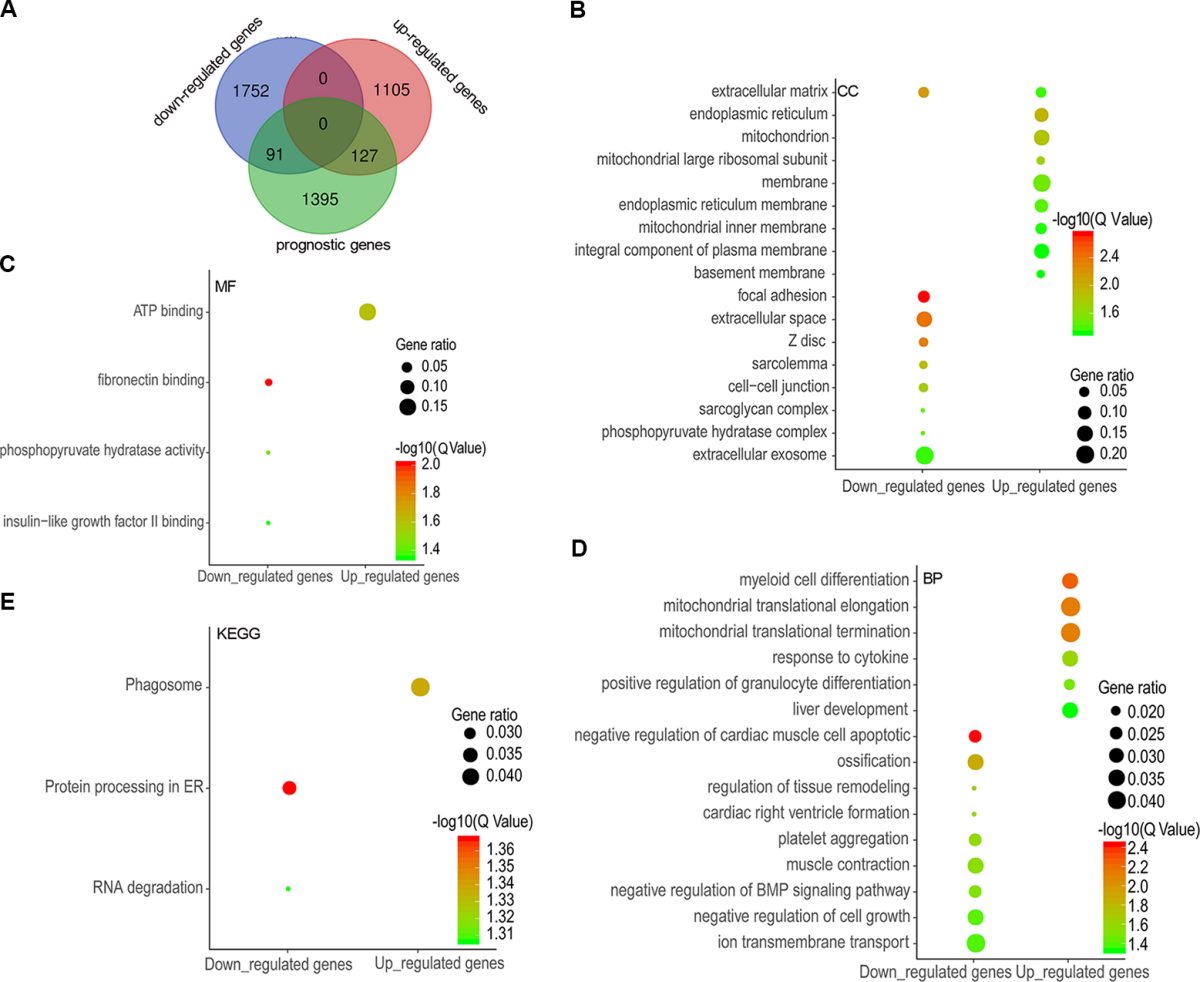
Differentially expressed genes (DEGs) overlapping with prognostic genes and functional enrichment analysis. A total of 91 downregulated genes and 127 upregulated genes were overlapped with potential prognostic genes **(A)**, and the enrichment results of cellular components **(B)**, molecular functions **(C)**, biological processes **(D)**, and KEGG **(E)** were visualized as gene ontology (GO) enrichment plot by ImageGP.

A total of 1,613 prognostic genes were identified in COAD by GEPIA 2, which comprised both overall survival (OS) and recurrence-free survival (RFS) genes (**Supplementary Table 2**). We further identified 218 prognostic genes overlapping with DEGs (91 downregulated and 127 upregulated genes) by intersecting prognostic genes and DEGs (**Figure 2A** and **Supplementary Table 3**), and were implemented enrichment analysis by DAVID Bioinformatics Resources 6.8. Cellular component (CC) results showed that 91 genes encoding proteins were mainly located outside the cell and 127 genes encoding proteins mainly located in the endoplasmic reticulum, mitochondrion, and membrane (**Figure 2B**). Molecular function (MF) results showed that 91 genes mostly performed function of fibronectin binding as well as phosphopyruvate hydratase activity and 127 genes mostly performed ATP binding (**Figure 2C**). As for biological process, 91 genes participated in the regulation of tissue remodeling, BMP signaling pathway, and cell growth, and 127 genes took part in mitochondrial translational elongation and termination as well as response to cytokine, etc. (**Figure 2D**). According to the Kyoto Encyclopedia of Genes and Genomes (KEGG) results, 91 genes were enriched in pathway of protein processing in endoplasmic reticulum (ER) and RNA degradation and 127 genes were enriched in phagosome pathway (**Figure 2E**).

### Fitness Genes in COAD Cells and Genes of Interest for Further Analysis

**Figure 3:**
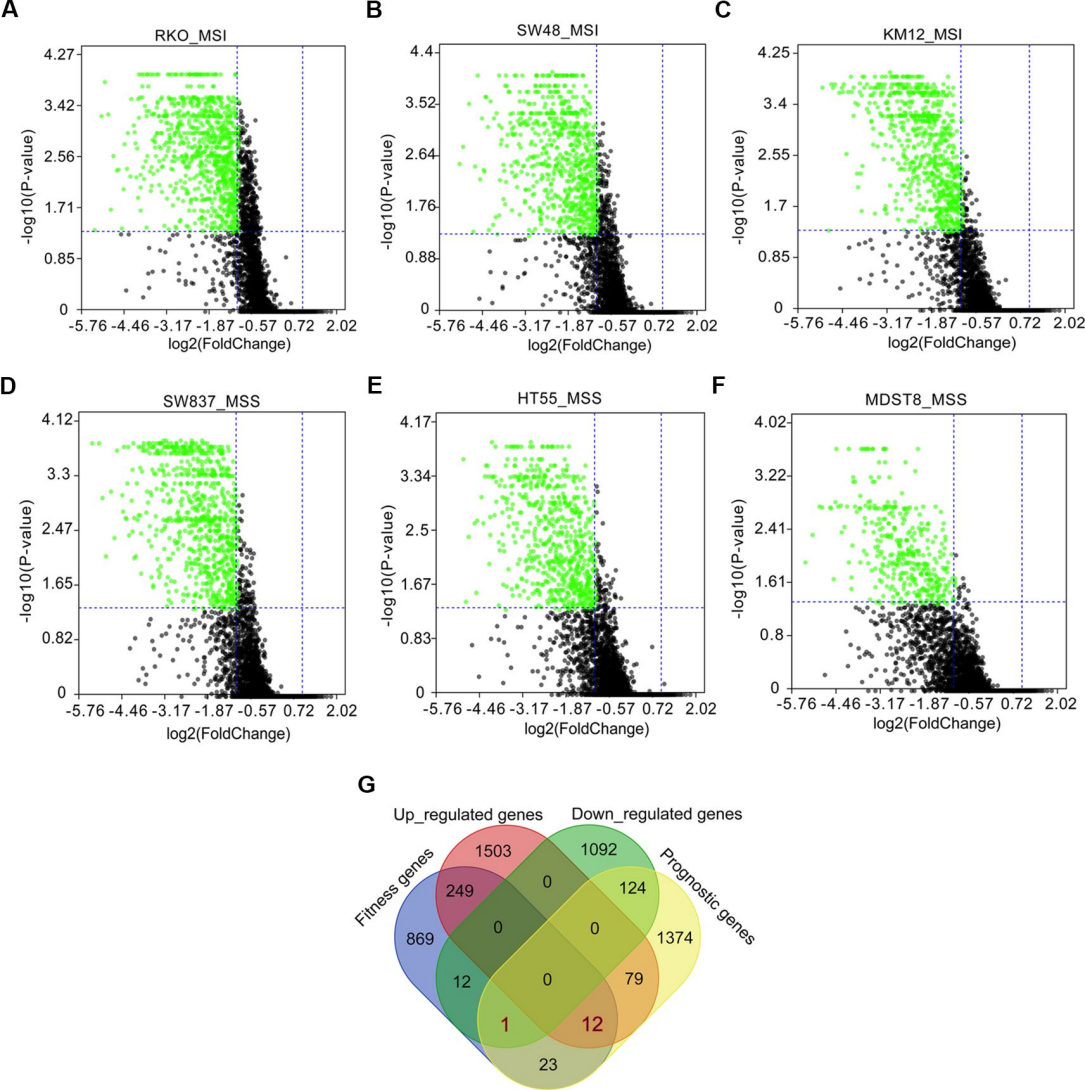
Fitness genes overlapping with prognostic genes and differentially expressed genes (DEGs) in colon adenocarcinoma (COAD). Fitness genes were defined as genes required for cell growth or viability (Behan et al., 2019). Genome-scale CRISPR-Cas9 knockout (GeCKO) screening results mining showed fitness genes (*green scatters*) in COAD cells with microsatellite instability (MSI), including RKO **(A)**, SW48 **(B)**, and KM12 **(C)**, as well as COAD cells with microsatellite stability (MSS), such as SW837 **(D)**, HT55 **(E)**, and MDST8 **(F)**. A total of 12 upregulated and one downregulated genes were discovered to overlap with prognostic and fitness genes **(G)**.

Fitness genes were defined as genes required for cell growth or viability (Behan et al., 2019), which were screened by GeCKO library in 324 human cancer cell lines including COAD cells with microsatellite instability (MSI) and microsatellite stability (MSS) (Behan et al., 2019). Fitness genes in COAD cell lines of RKO, SW48, KM12, SW837, HT55, and MDST8 were visualized as volcano plots (**Figures 3A–F**), and 1,166 genes promoting the growth of at least three cell lines were set as fitness genes in COAD (**Figure 3G** and **Supplementary Table 4**).

A total of 12 upregulated and one downregulated genes were found to overlap with prognostic and fitness genes (**Figure 3G**), in which four upregulated genes (*TUBA1C*, *ABCE1*, *UBE2N*, and *NIFK*) had been reported to play roles in tumor growth, cell cycle, migration, metastasis, and prognosis (**Supplementary Table 5**). So, the remaining eight upregulated as well as one downregulated genes were regarded as target genes for further analysis in the following study (**Supplementary Table 5**).

### Expression of Target Genes in COAD and Its Subtypes

**Figure 4:**
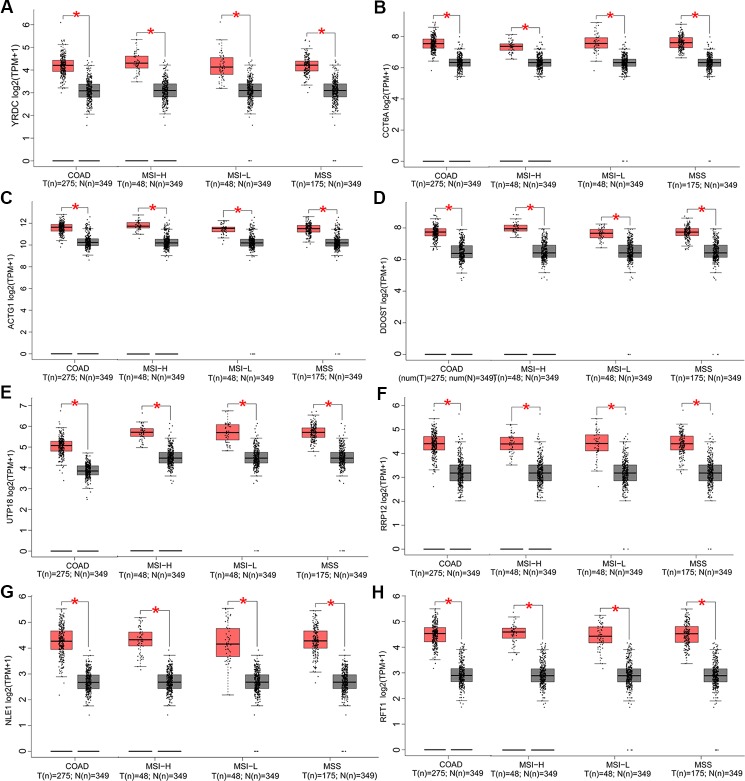
Expression of the eight upregulated genes in colon adenocarcinoma (COAD) and its subtypes. Expressions of *YRDC* (*P* < 0.01) **(A)**, *CCT6A* (*P* < 0.01) **(B)**, *ACTG1* (*P* < 0.01) **(C)**, *DDOST* (*P* < 0.01) **(D)**, *UTP18* (*P* < 0.01) **(E)**, *RRP12* (*P* < 0.01) **(F)**, *NLE1* (*P* < 0.01) **(G)**, and *RFT1* (*P* < 0.01) **(H)** in COAD and its subtypes with microsatellite instability—high (MSI-H), microsatellite instability—low (MSI-L), or microsatellite stability (MSS)

**Figure 5:**
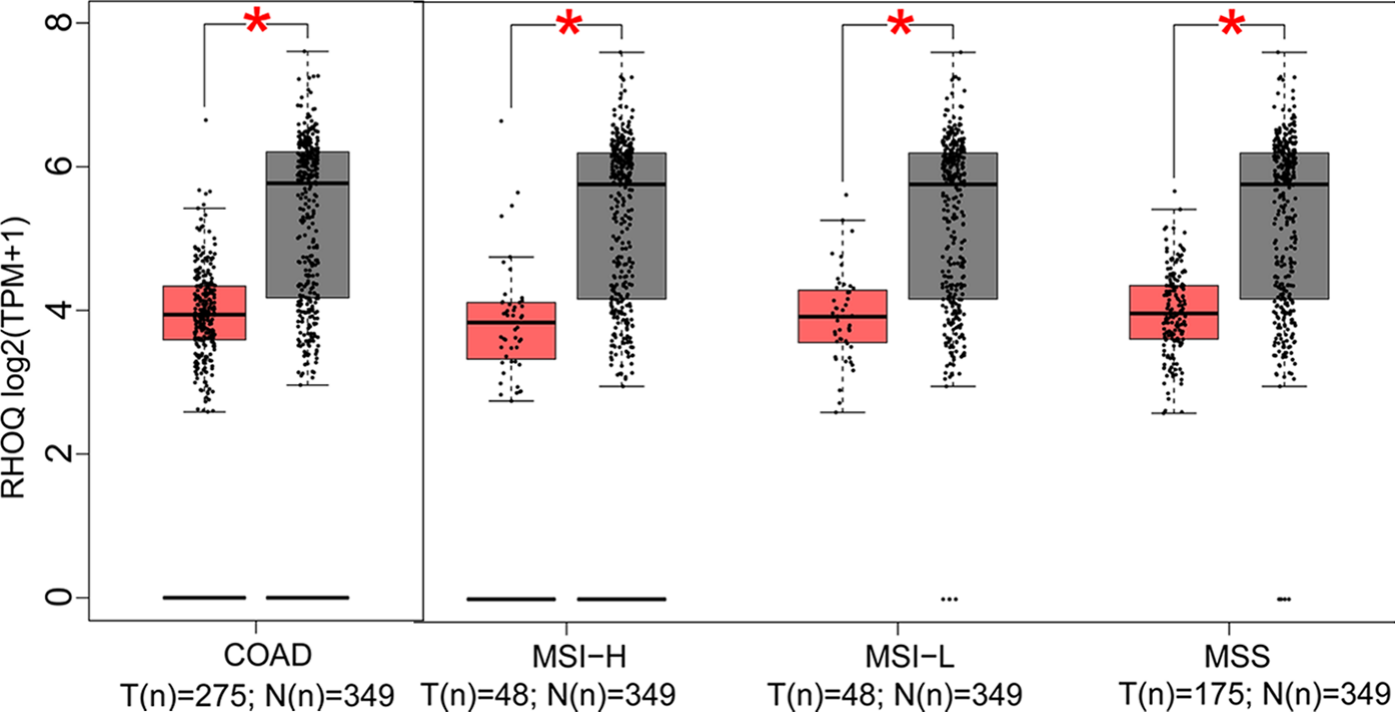
Expression of the downregulated gene in colon adenocarcinoma (COAD) and its subtypes. Expression of *RHOQ* (*P* < 0.01) in COAD and its subtypes with microsatellite instability—high (MSI-H), microsatellite instability—low (MSI-L), or microsatellite stability (MSS).

In this part, we analyzed the expression of the nine target genes in COAD by GEPIA 2. Expression of the eight upregulated genes (*YRDC*, *CCT6A*, *ACTG1*, *DDOST*, *UTP18*, *RRP12*, *NLE1*, as well as *RFT1*) increased significantly in COAD and its subtypes (**Figures 4A–H**) and the expression of *RHOQ* decreased significantly in COAD and its subtypes (**Figure 5**). While expression of the above genes among COAD subtypes did not show significant difference (**Supplementary Figures 1A–I**).

### Prognostic Significance of Target Genes in COAD and Its Subtypes

**Figure 6:**
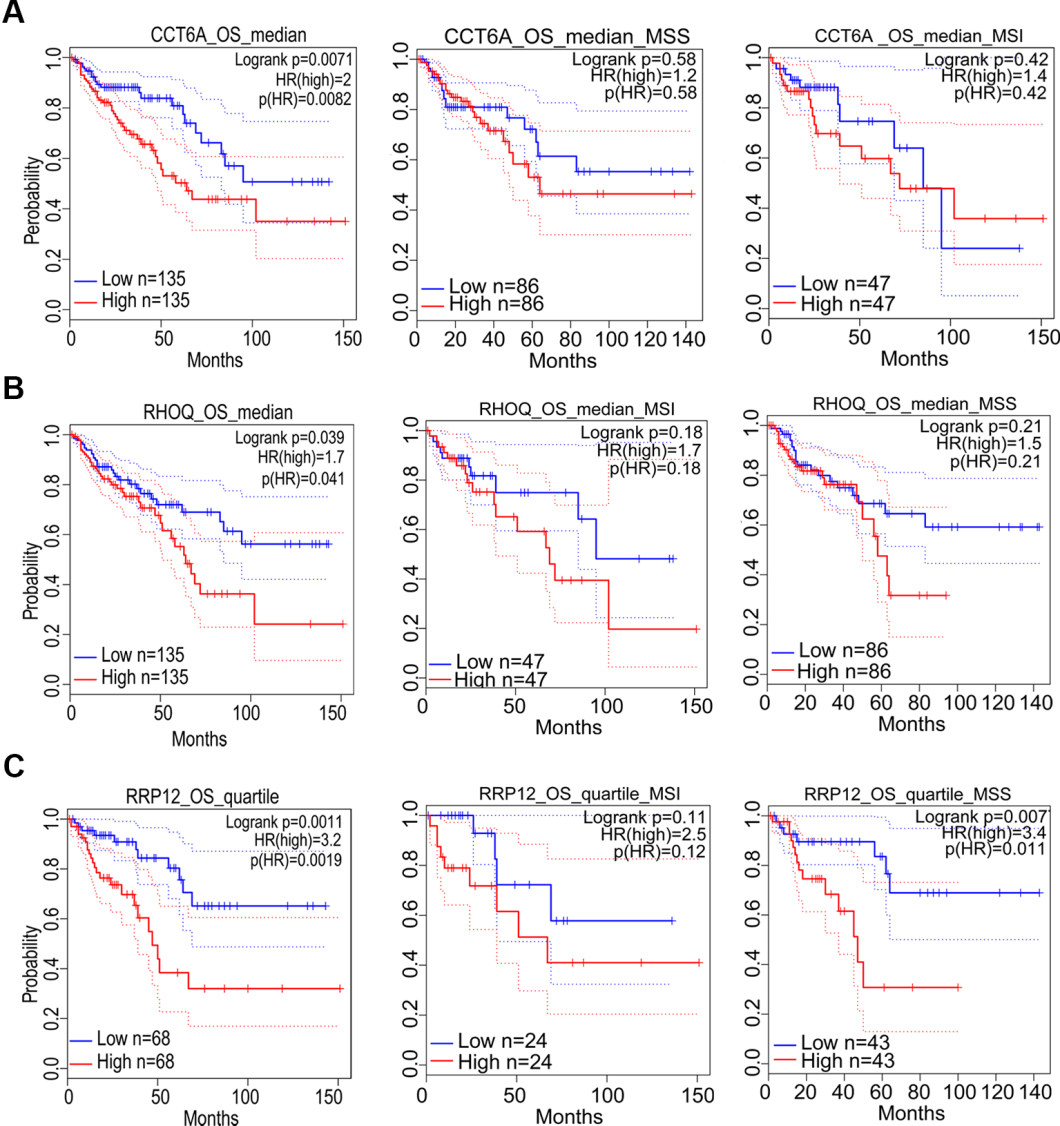
The correlation between gene expression and poor prognosis in colon adenocarcinoma (COAD) and its subtypes (TCGA data). The correlation between high expression of *CCT6A* **(A)**, *RHOQ* **(B)**, as well as *RRP12* **(C)** and survival rates in COAD and its subtypes.

**Figure 7:**
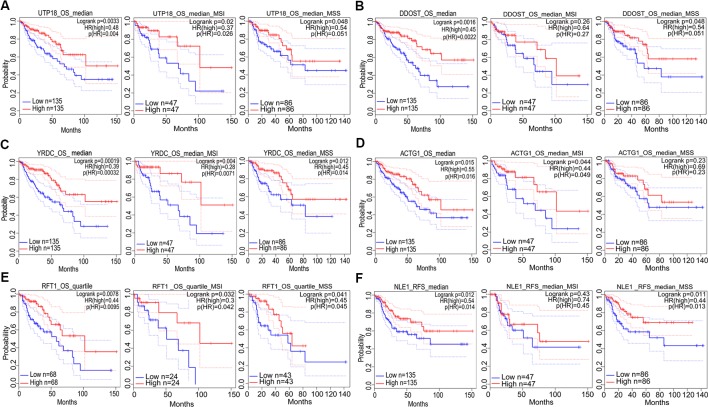
The correlation between gene expression and good prognosis in colon adenocarcinoma (COAD) and its subtypes (TCGA data). The correlation between high expression of *UTP18* **(A)**, *DDOST* **(B)**, *YRDC* **(C)**, *ACTG1* **(D)**, *RFT1* **(E)**, as well as *NLE1* **(F)** and survival rates in COAD and its subtypes.

Prognostic significance of the nine target genes in COAD were analyzed by GEPIA 2. High expressions of *CCT6A*, *RHOQ*, and *RRP12* were relative to lower survival rates of COAD patients and its subtypes (**Figures 6A–C**). High expressions of *UTP18*, *DDOST*, *YRDC*, *ACTG1*, *RFT1*, and *NLE1* were relative to higher survival rates of COAD patients or its subtypes (**Figures 7A–F**).

### Effects of Gene Knockout on COAD Cell Growth

**Figure 8:**
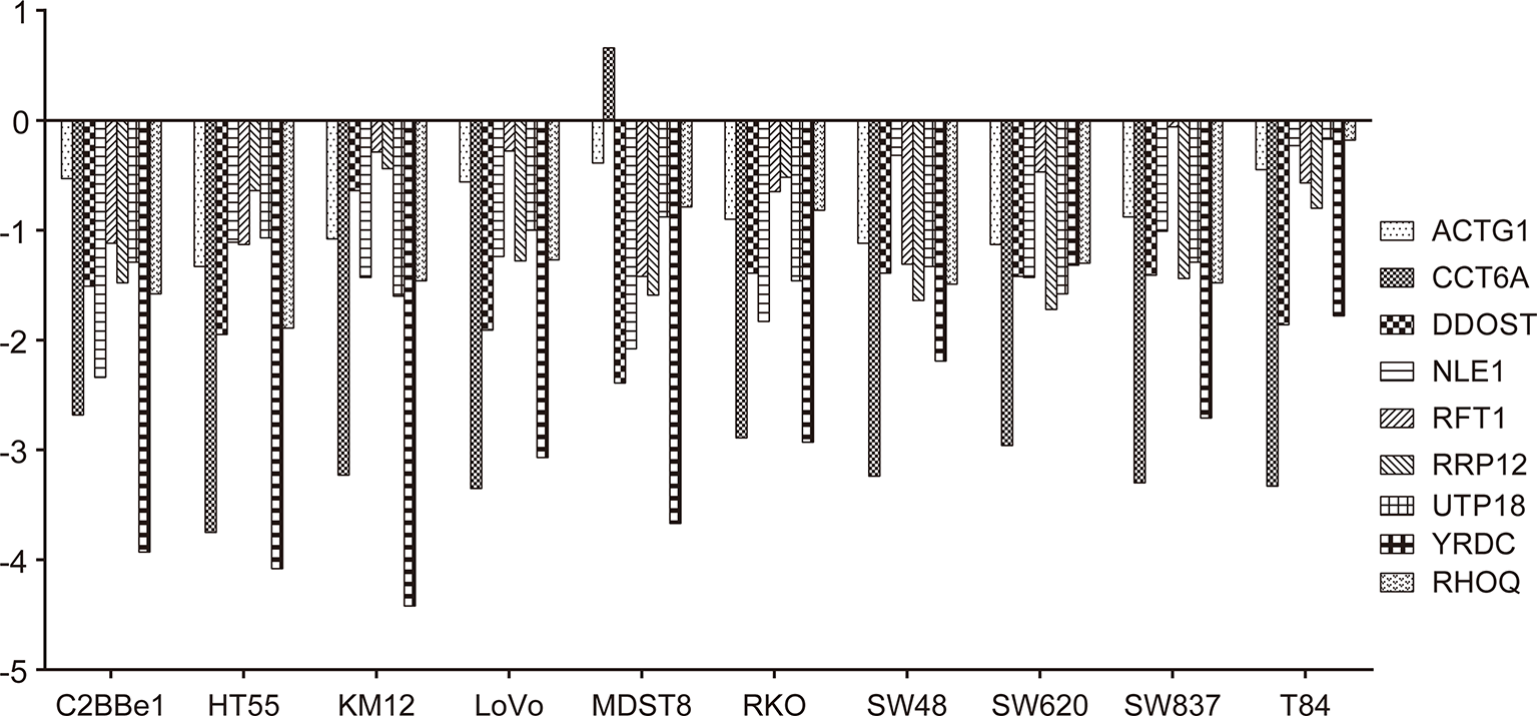
Effects of gene knockout on colon adenocarcinoma (COAD) cell growth. Effects of nine target genes depletion on COAD cell growth or viability. Log_2_FC < −1 indicates that cell proliferation or viability was inhibited by gene deletion. All results were mined GeCKO data downloaded from the project Score web portal (https://score.depmap.sanger.ac.uk)

Deletion of the nine target genes all inhibited growth and viability of some COAD cell lines according to GeCKO screening data (**Figure 8**).

### Dependency Score of Target Genes Across Pan-Cancer Cells

**Figure 9:**
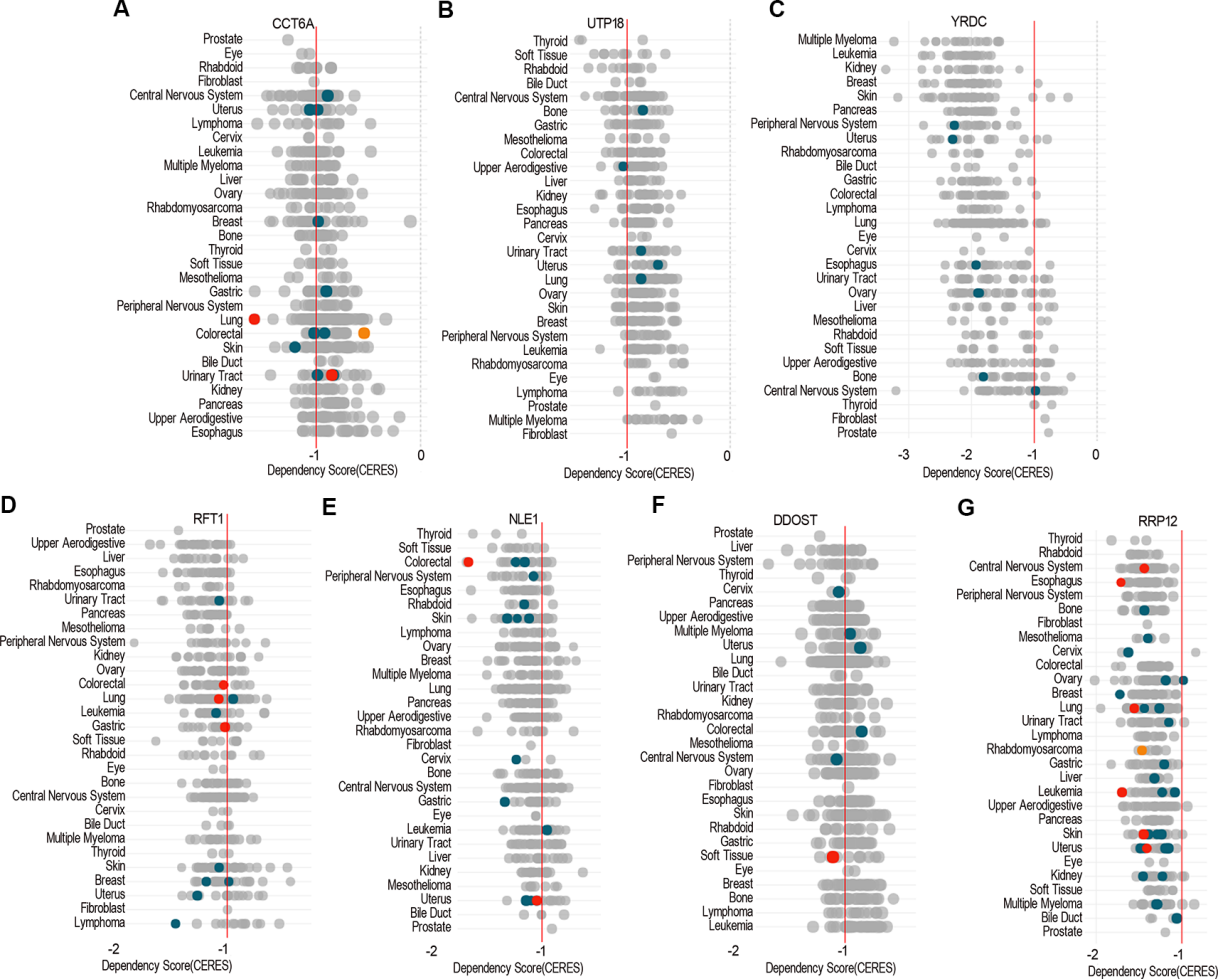
The essential genes across pan-cancer. Dependency scores of *CCT6A* **(A)**, *UTP18* **(B)**, *YRDC* **(C)**, *RRP12* **(D)**, *RFT1* **(E)**, *NLE1* **(F)**, and *DDOST* **(G)** across pan-cancer based on GeCKO screening data by DepMap web portal (https://depmap.org/portal/depmap). The CERES dependency score is based on data from a cell depletion assay. A lower CERES score indicates a higher likelihood that the gene of interest is essential in a given cell line. A score of 0 indicates a gene is not essential; correspondingly, −1 is comparable to the median of all pan-essential genes (*red line*). The *color* and *size of circles* represent mutation and expression, respectively.

Dependency score analysis by DepMap showed that *ACTG1* and *RHOQ* were less essential across pan-cancer cells including COAD cell lines (**Supplementary Figures 2A, B**), and *CCT6A*, *UTP18*, *YRDC*, *RRP12*, *RFT1*, *NLE1*, as well as *DDOST* were essential across pan-cancer cells including most of COAD cell lines (**Figures 9A–G**).

## Discussion

Colon and rectal cancers had remarkably similar patterns of genomic or epigenetic alterations, excluding 16% hypermutated tumors (Cancer Genome Atlas 2012). The Cancer Genome Atlas (TCGA), a public-funded project that aims to create a comprehensive “atlas” of cancer genomic profiles (Tomczak et al., 2015), recorded 382 samples of colorectal adenocarcinoma with RNA sequencing (RNA-Seq) data, and we mined DEGs in 275 COAD patients compared with 41 TCGA normal and 308 Genotype–Tissue Expression (GTEx) colon tissues by GEPIA 2 (Tang et al., 2017). In consideration of integrating more normal RNA-Seq data, we got similar but different DEGs in COAD (Xi et al., 2017; Long et al., 2018). Enrichment analysis of KEGG by GSEA (Subramanian et al., 2005) showed that the downregulated genes mainly participated in DNA replication, cell cycle, ribosome pathway, etc., and the upregulated genes mainly participated in vascular smooth muscle contraction, dilated cardiomyopathy, calcium signaling pathway, etc. In addition, Hallmarks and oncogenic signatures enrichment results showed that the downregulated genes were mainly enriched in MYC targets, MTORC1 signaling, glycolysis, SINGH KRAS DEPENDENCY SIGNATURE, P53 DN.V1 UP, etc., and the upregulated genes mainly enriched in epithelial mesenchymal transition, PTEN DN.V1 UP, P53 DN.V1 DN, CYCLIN D1 KE V1 DN, etc.

Screening prognostic genes could distinguish patients with high risks from those with low risks for COAD recurrence and predicting prognosis of patients (Xu et al., 2016; Xu et al., 2017). Additionally, clinical prognostic correlation could further imply the gene would play an important role in tumorigenesis and development. We performed survival analysis including OS and RFS based on the expression status of every gene by GEPIA 2 (Tang et al., 2017) and obtained a list of the most significant survival-associated genes overlapping with the top DEGs in COAD. Enrichment analysis of GO and KEGG was conducted and showed that these prognostic genes mainly took part in cell growth, mitochondrial translational elongation and termination, as well as response to cytokine and were enriched in pathways of protein processing in ER, RNA degradation, and phagosome.

In order to reveal the possible mechanism of prognostic function of DEGs, we mined the GeCKO screening data of 324 human cancer cell lines including COAD cells with MSI and MSS (Behan et al., 2019) to get a fitness gene list in COAD. A total of 12 upregulated and one downregulated genes overlap with prognostic and fitness genes. After reviewing the literature, we found that four upregulated genes comprising of *TUBA1C* (Li et al., 2017; Wang et al., 2017), *ABCE1* (Hlavata et al., 2012; Tian et al., 2016), *UBE2N* (Kim et al., 2015; Dikshit et al., 2018), and *NIFK* (Pan et al., 2015; Lin et al., 2016) had been reported to function in tumor growth, proliferation, migration, metastasis, and prognosis. Therefore, we next focused on the analysis of the remaining nine target genes.

COAD patients with MSI and MSS usually have different clinical outcomes (Kawakami et al., 2015) and efficacy of adjuvant 5-fluorouracil chemotherapy (Zaanan and Taieb 2019), which reflected the heterogeneity of COAD. Expression of the eight upregulated genes (*YRDC*, *CCT6A*, *ACTG1*, *DDOST*, *UTP18*, *RRP12*, *NLE1*, as well as *RFT1*) increased both in COAD and its subtypes compared with normal, while the expression of *RHOQ* decreased both in COAD and its subtypes. However, expression of target genes among COAD subtypes showed no significant difference. The above results implied that the target genes participated in the development or progression of COAD, not specifically in subtypes with MSI or MSS.

In addition, prognostic significance analysis demonstrated that high expressions of *CCT6A*, *RHOQ*, and *RRP12* were relative to lower survival rates (OS or RFS) in COAD patients or subtypes, which could be interpreted as promoting growth or viability of COAD cells because deletion of each of the above genes inhibited the growth of at least three COAD cell lines according to our fitness gene mining results. However, high expressions of *UTP18*, *DDOST*, *YRDC*, *ACTG1*, *RFT1*, and *NLE1* were relative to higher survival rates (OS or RFS) in COAD patients or subtypes, although their depletion inhibited the growth of some COAD cell lines. This result seemed to be contradictory, but it was reasonable. First, clinical prognostic factors of COAD included malignant degree, tumor stage, age, patient psychological quality, sample size, follow-up time, etc. It was difficult to determine the prognostic result according to the expression of one gene. Second, the protein or RNA expressed by each gene exerts cellular functions in a network regulation mode, and each gene might perform multiple functions. We only analyzed the effects of target genes on cell growth, but did not rule out that the same gene performed other functions during tumor progression and prognosis. Third, tumors were highly heterogeneous (Kawakami et al., 2015; Pan et al., 2016; Kandagatla et al., 2018), and the same gene might play different roles in different tumor samples.

Lastly, pan-cancer analysis of dependency score (Meyers et al., 2017; Tsherniak et al., 2017) showed that *CCT6A*, *UTP18*, *YRDC*, *RRP12*, *RFT1*, *NLE1*, as well as *DDOST* were essential across pan-cancer including COAD cells, while *ACTG1* or *RHOQ* were less essential in cancer cells, which indicated that the essential genes would be developed into anticancer drugs as potential targets.

In summary, we identified nine DEGs associated with prognosis and growth in COAD based on integrated bioinformatics analysis. High expressions of *CCT6A*, *RRP12*, and *RHOQ* promoted growth of COAD cell lines and were relative to poor prognosis, while high expressions of *UTP18*, *DDOST*, *YRDC*, *ACTG1*, *RFT1*, and *NLE1* were relative to good prognosis, although they promoted growth of some COAD cells. In the end, *CCT6A*, *UTP18*, *YRDC*, *RRP12*, *RFT1*, *NLE1*, and *DDOST* were essential genes across pan-cancer including COAD cells. In a word, we found nine novel potential anticancer targets and prognostic markers in COAD and its subtypes. The limitations of this study were lack of cytological functional experiments as well as molecular mechanism research of target genes, which was the direction of our future work.

## Data Availability Statement

All datasets generated for this study are included in the article/**Supplementary Material**.

## Author Contributions

HZ, XD, and JS designed the study. MH, XF, and ZS mined and interpreted the data. CL provided clinical support. HZ, MH, and XF wrote the manuscript. All authors reviewed and approved the final manuscript.

## Funding

This study is funded by the Natural Science Research Project of Jiangsu Higher Education Institutions (18KJB310002), scientific research project of Jiangsu Provincial Health and Family Planning Commission (Y2018101), Heilongjiang Provincial Health Department Project (2017-406, no. 2018-383 and 2018-509), and the Science and Technology Innovation Team Construction Project of Jiangsu Vocational College of Medicine (20188104).

## Conflict of Interest

The authors declare that the research was conducted in the absence of any commercial or financial relationships that could be construed as a potential conflict of interest.

## Supplementary Material

Supplementary Figure 1 Expression of the target genes among COAD subtypes.

Supplementary Figure 2 The less essential genes across pan-cancer.

Supplementary Table 1 DEGs in COAD.

Supplementary Table 2 Potential prognostic genes in COAD.

Supplementary Table 3 DEGs overlapping with prognostic genes.

Supplementary Table 4 Fitness genes in COAD.

Supplementary Table 5 DEGs overlapping with prognostic and fitness genes.
