# Supplementary figures and images for: Identification of Differently Expressed Genes Associated With Prognosis and Growth in Colon Adenocarcinoma Based on Integrated Bioinformatics Analysis
Source: Front Genet. 2019 Dec 4;10:1245. doi: 10.3389/fgene.2019.01245 (PMC6905401; doi:10.3389/fgene.2019.01245)

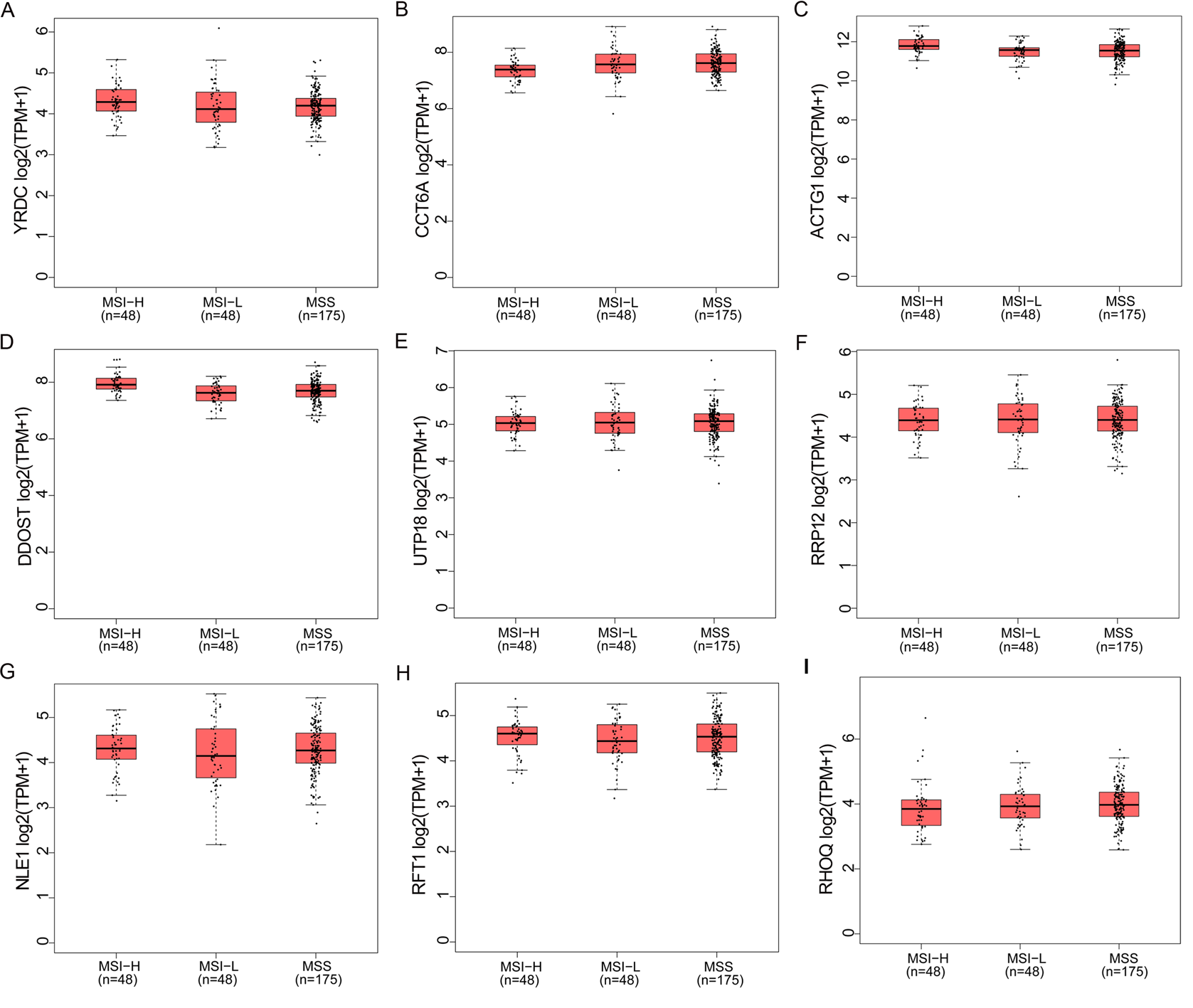

Supplement: Supplementary Figure 1 — Expression of the target genes among COAD subtypes. [file Image_1.tif]

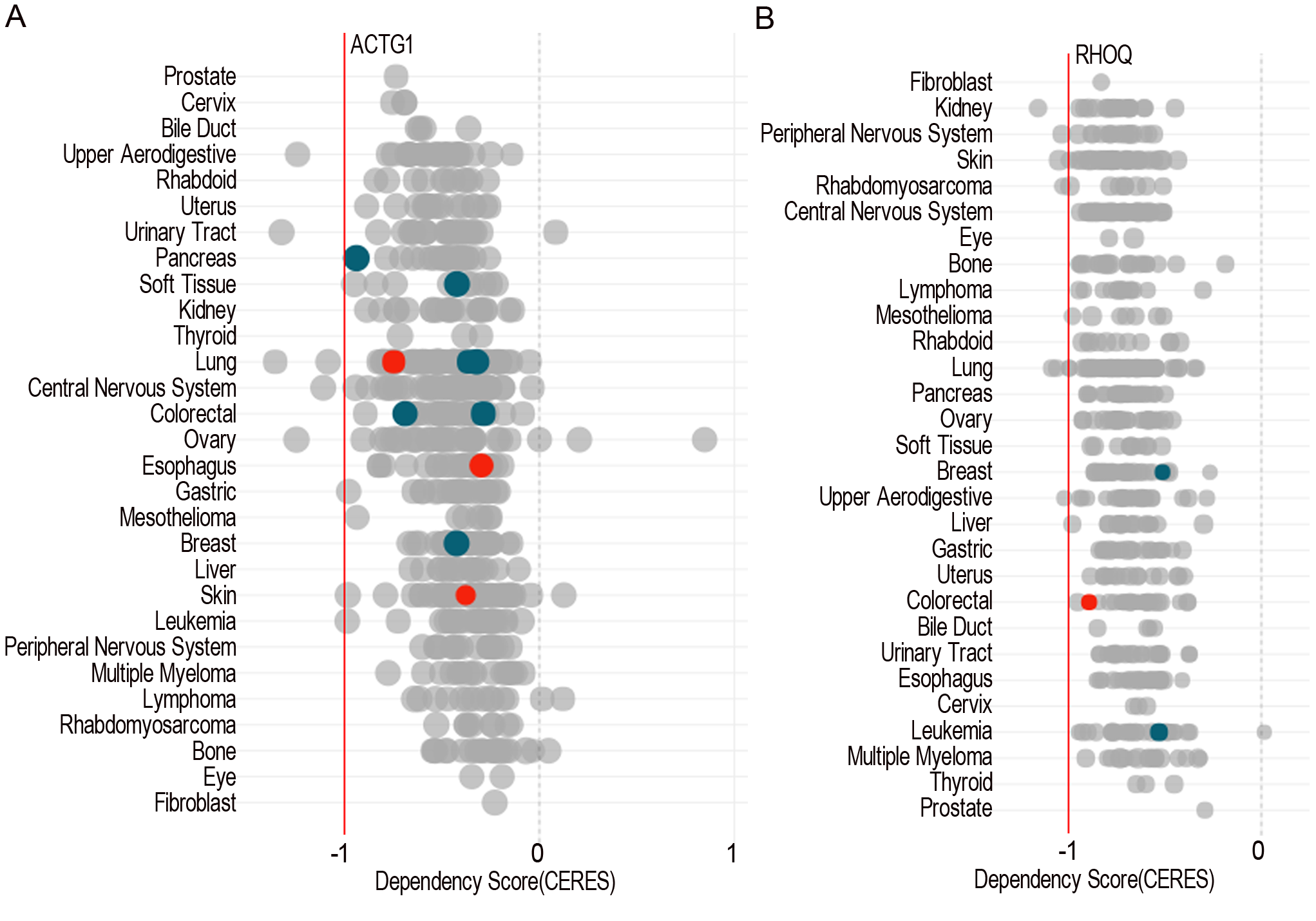

Supplement: Supplementary Figure 2 — The less essential genes across pan-cancer. [file Image_2.tif]
