# Supplementary material for: Identification of Differently Expressed Genes Associated With Prognosis and Growth in Colon Adenocarcinoma Based on Integrated Bioinformatics Analysis
Source: Front Genet. 2019 Dec 4;10:1245. doi: 10.3389/fgene.2019.01245 (PMC6905401; doi:10.3389/fgene.2019.01245)
Supplement: Supplementary Table 5 — DEGs overlapping with prognostic and fitness genes. [file Table_5.docx]

**Supplementary Table 5. DEGs overlapping with prognostic and fitness genes in COAD**

| Gene | Location in cell | Reference in tumors | Function |
| --- | --- | --- | --- |
| TUBA1C | Cytoskeleton, nucleus, cytosol | 1 | Prognosis, Proliferation, Migration |
| ABCE1 | Mitochondrion, cytosol, nucleus | 18 | Proliferation, Migration, Metastasis, Apoptosis |
| UBE2N | Nucleus, cytosol, mitochondrion | 5 | Cell death, Growth. |
| NIFK | Nucleus, cytosol | 3 | Prognosis, Proliferation, Cell cycle. |
| ACTG1 | Extracellular, cytoskeleton, cytosol | 2 |  |
| CCT6A | Cytoskeleton, cytosol | 3 |  |
| YRDC | Mitochondrion, extracellular | 2 |  |
| DDOST | Endoplasmic reticulum, plasma membrane, Lysosome | 0 |  |
| UTP18 | Nucleus, cytosol | 0 |  |
| RFT1 | Endoplasmic reticulum, plasma membrane | 1 |  |
| RRP12 | Nucleus, mitochondrion | 0 |  |
| NLE1 | Nucleus, cytosol | 0 |  |
| RHOQ | Plasma membrane, cytoskeleton, cytosol | 1 |  |

Note: Red indicates up-regulated genes, green indicates down-regulated genes.
